# Supplementary material for: Cancer patient survival can be parametrized to improve trial precision and reveal time-dependent therapeutic effects
Source: Nat Commun. 2022 Feb 15;13:873. doi: 10.1038/s41467-022-28410-9 (PMC8847344; doi:10.1038/s41467-022-28410-9)
Supplement: Supplementary file 5 — Supplementary Dataset 2 [file 41467_2022_28410_MOESM5_ESM.zip › Supplementary Data File 2/Delta alpha vs beta plots- Fig 6, 7, S9/Heatmap censoringb4 n100 k1000.pdf]

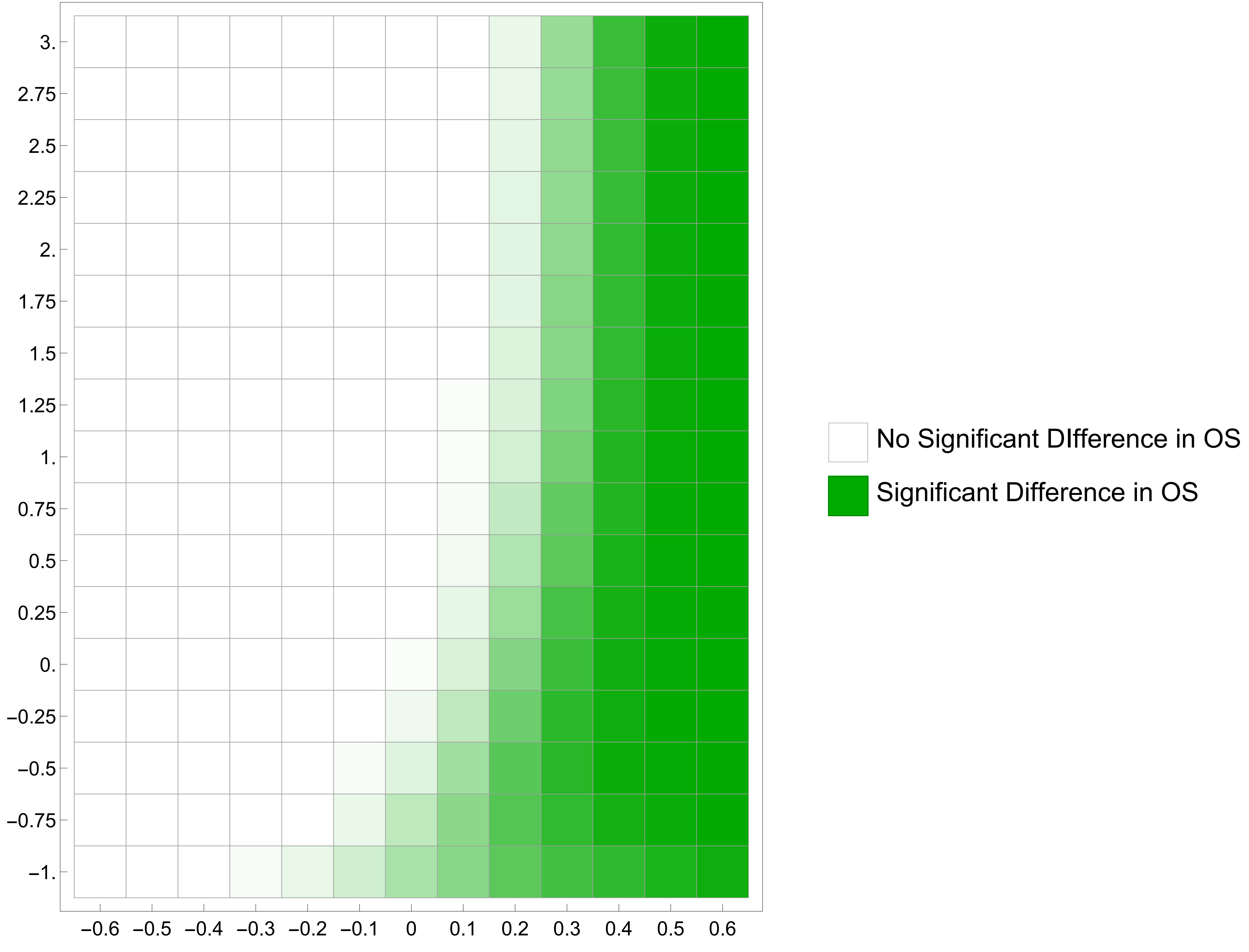

3.
2.75
2.5
2.25
2.
1.75
1.5
1.25
1.
0.75
0.5
0.25
0.
−0.25
−0.5
−0.75
−1.
−0.6
−0.5
−0.4
−0.3
−0.2
−0.1
0
0.1
0.2
0.3
0.4
0.5
0.6
No Significant DIfference in OS
Significant Difference in OS
